# Provider-perceived benefits and constraints of complete adherence to antenatal care guideline among public health facilities, Ethiopia: A qualitative study

**DOI:** 10.1371/journal.pone.0255297

**Published:** 2021-08-09

**Authors:** Tewodros Seyoum, Mekuriaw Alemayehu, Kyllike Christensson, Helena Lindgren

**Affiliations:** 1 School of Midwifery, College of Medicine Health Sciences, University of Gondar, Gondar, Ethiopia; 2 Department of Environmental Health, Institute of Public Health, University of Gondar, Gondar, Ethiopia; 3 Department of Women’s and Children’s Health, Karolinska Institutet, Stockholm, Sweden; Agha Khan University, UNITED REPUBLIC OF TANZANIA

## Abstract

**Background:**

In Ethiopia, health care providers’ level of adherence to the national Antenatal Care (ANC) guideline is relatively low. The reasons why they do not follow the guidelines are not well known. Therefore, this study aimed to explore the provider-perceived benefits and constraints associated with using the guideline for ANC in public health facilities in Gondar town.

**Methods:**

A qualitative study was conducted using a semi-structured interview guide. The interview was conducted among a purposive sample of nine health care providers working in four public health facilities in Gondar town. After the interviews were transcribed and coded, a content analysis was done using Atlas ti version 7.5 software packages.

**Result:**

Decreasing provider’s workload and maximizing performance, improving safe motherhood, and improving the process of service delivery were reported as the perceived benefits of following ANC guideline. Organizational problems, care providers’ existing knowledge, attitude, and skills and availability of training and mentorship were the three main identified groups of factors that hinder complete providers’ adherence to ANC guideline.

**Conclusion:**

Although providers acknowledged the benefits of following ANC guideline, the guideline is not fully implemented. Refresher training should be given at the start of the updated eight-contact ANC guideline and continuing education and supervision throughout the implementation process. Health care providers call for profound and urgent revisions of the supply chain system for supplies and equipment.

## Background

Worldwide, improving the quality of care using the best available evidence is a major challenge facing the health care system [1, 2]. Clinical guidelines are high-level evidence-based tools designed to improve the quality of care [3]. Using guidelines during clinical practice has several benefits for both the provider and end service users. These benefits include improving client outcomes, ensuring uniformity of care, improving the process and structure of care, and helping providers to make clinical decisions [4–8].

Development and dissemination of guidelines in the clinical setting alone can’t improve the quality of care unless the guidelines are effectively implemented and fully adhered to [9]. There are several reasons why providers fail to follow all guidelines. These include scarcity of resources, leadership, organizational culture differences, lack of training and supervision, limited familiarity with current clinical guidelines, and resistance on the part of some providers to accept new recommendations [5, 10, 11].

Antenatal care (ANC) is one of evidence-based interventions flagged internationally to improve Maternal and Child Healthcare (MCH) [12]. Since 2002, the World Health Organization (WHO) four-visit focused ANC guideline has been implemented in Low and Middle-Income Countries (LMIC) [13]. However, recently concerns have been raised that a reduced number of visits is associated with an increase in stillbirth [14]. Based on these findings, WHO developed a new eight-contact ANC guideline in 2016, which aims to improve the quality of ANC [15]. However, in many countries worldwide, including Ethiopia, the four visits focused ANC continues to be followed as the standard practice [13].

It’s easy to say that in the last few decades a great deal of effort has been made to generate evidence, with the final objective to improve the quality of maternal care. The guidelines for ANC are based on best available evidence [15, 16]. However, in many of LMICs, turning evidence-based focused ANC guideline into clinical practice is a missing link [17]. The potential class between a standardized practice as described in ANC guideline and providing care according to women’s needs should be strengthened. Pregnant women may need emotional, psychological, and social support in addition to the basic ANC service [15].

In Ethiopia, it is palpable that maternal and neonatal mortality is still unacceptably high though currently there are indications of a marked decline. According to the 2016 Ethiopian demographic health survey (EDHS), the maternal mortality rate was 412/100,000 live births [18]. Reducing maternal mortality and improving maternal health is a top priority of the Ethiopian health authorities by increasing the coverage of focused ANC with at least four visits [19]. As one of the low-income countries, Ethiopia has accepted and been implementing focused ANC [20]. From 2005 to 2019, the proportion women attending first ANC visit increased from 28 to 74% with disparity among regions [18, 21]. The provision of ANC services in urban areas of Ethiopia is the joint responsibility of midwives and obstetricians [22].

The history of working with ANC guideline is not new in Ethiopia. However, the guideline is vage with no clear independent document used as focused ANC guideline [23]. the ANC guideline is embedded into the Ethiopian national obstetric protocol and incorporated in Basic Emergency Obstetric Newborn Care (BEmONC) training manuals [24, 25]. As a national consensus, these official documents are used as focused ANC guideline. Within these national documents, an integrated antenatal, labor, delivery, newborn and postnatal Care card is included and used as a working document during clinical practice. A local study revealed that lack of clear ANC guideline resulted in poor quality of care and broken trust between client and health care providers [23].

More than one in four (26%) Ethiopian women do not receive any ANC visits at all, and even those who receive it do not get quality services due to providers’ inability to adhere to focused ANC guideline [18, 26]. As an example, a cross-sectional study done in Gondar town indicated that only 32% of women received complete providers’ adherence to national ANC guideline during the first visit [27]. However, the reasons for the poor adherence to ANC guideline are not well known. It was crucial to understand and learn more from the health care providers themselves about their reasons for not following the guideline.

Therefore, this study aimed to explore the provider-perceived benefits and constraints associated with complete adherence to ANC guideline among public health facilities in public health facilities in Gondar town.

## Methods and materials

### Study design, period and setting

A qualitative study was conducted in Gondar town public health facilities in December 2019. The study was conducted in one comprehensive specialized hospital and three public health centers. According to the Ethiopian health tier system, health centers are classified under the level of primary healthcare and specialized hospitals are categorized under tertiary level of healthcare [28]. An average of 100–150 clients visit the University of Gondar specialty hospital everyday for ANC service, and 20–30 ANC users visit each health center every day.

### Recruitment of study participants

Participants in this study were health care providers working in four public health facilities. A purposive sampling was employed to select health care providers who were actively working in ANC units and took into account their specialties and experience [29]. Among the selected, nine health care providers participated in the study; two of them were obstetricians with sub-specialties and the remaining seven were midwives. Two of the midwives had masters in clinical midwifery, five had bachelor degrees, and two had diploma in midwifery. The mean clinical experience of the participants was five years.

### Data collection process

First, an interview guide (S1 Text) and manual were prepared by the principal investigator to make sure that the same points were covered during every interview. The guide was developed in English and translated into Amharic (the local language). An invitation letter was sent to the participants to fix the date and time of the interview.

After securing written informed consent, face-to-face interviews were conducted by two investigators with participants to explore how they perceived the benefits and bottlenecks of adhering to ANC guideline. The interviews were conducted within the participants’ workplace.

The interview was initiated by presenting general open-ended questions about the reason for following a set of guidelines. This was done in an effort to establish a rapport with the participants. Then set of questions related with the benefits and constraints of following ANC guideline were presented. After these questions had been answered, the interviews continued by asking open-ended questions based on each individual’s response. The data collection was completed after nine individual interviews when no new/unique information emerged [30].

Interviews were audio recorded using digital devices and notes were taken. The interviews were between 20 and 40 minutes long.

### Data management and analysis

Upon completion of each interview, the recorded audio was transcribed into the Amharic language, and these transcripts were then translated into English by an expert fluent in both languages and made available in Microsoft word files for analysis.

The translated and transcribed data were exported and analyzed by content analysis method using ATLAS.ti version 7 software [31, 32]. The analysis was conducted jointly by all investigators.

The analysis went through the following steps: First, the transcribed text was carefully read several times in order to get the general sense of the content. Then general and condensed meaning units were identified. Codes were assigned to the condensed meaning units and discrepancies in the coding were discussed and a consensus was found. After this open coding, sub-categories were created by merging those with codes that were similar in meaning. Furthermore, sub-categories were combined to establish categories based on proximity of ideas. In the last phase, a general description of the research topic was formulated through generated categories.

### Ethical considerations

The protocol was reviewed by the IRB of the University of the Gondar and it was found to be ethically acceptable with study **ID. No: O/V/P/RCS/05/498/2018**. Before the start of the interviews, all the study participants gave their written informed consent. The interviewers had informed the participants about the purpose of the study and guaranteed the confidentiality of their data.

## Results

The data give us information about each participant’s perception of the benefits and the constraints for adhering to ANC guideline. We found that the perceived benefits of following the ANC guideline could be divided into three categories: I) Providers’ workload and performance; II) Improves perinatal outcomes and III) Improve service delivery (Table 1). We also found that there are three groups of factors that may hinder providers from following the ANC guideline: 1) Organizational problems; 2) Care providers’ existing knowledge, attitude, and skills and 3) Availability of training and mentorship (Table 2).

**Table 1:** Perceived benefits following ANC guideline during clinical practice in public health facilities in Gondar town.

Categories	Sub-categories
Providers’ workload and performance	Impact on time
	Structures the care activities
	Increases providers’ competence
Improve service delivery	Ensure the standard of care
	Enhance women-friendly care
	Improve continuity of care
	Improve the process of care
	Maintain the quality of service
Improves perinatal outcomes	Positive maternal outcomes
	Good fetal/neonatal outcomes
	Safe child birth

**Table 2:** Challenges that affect the complete adherence to ANC guideline.

Categories and sub-categories of constraints	Description of barriers
**Organizational constraints**	
Guideline related factors	Unavailability of guideline
	lacking details/depth of ANC sheet
	Lack of regular update of guideline
Human resource-related factors	High client flow/load
	Lack of manpower
	Miss- management of human power
Lack of appropriate facility set up	Inadequate and narrow ANC room
	Unavailability of separate MCH laboratory
	Irregular power supply
Logistic-related factors	Shortage of reagents and supplies
	Lack of laboratory machines
	Shortage of functional instruments (BP apparatus)
	Problems in the procurement process
	Lack of coordination between levels
**Care providers’ knowledge, attitude and skill**	
Poor providers’ attitude and behavior	Lack of habit to use guideline
	Lack of motivation and commitment
Lack of health providers’ knowledge and skill	Variation in providers’ knowledge of guideline
	Lack of providers’ experience in using guideline
	Lack of providers’ competency
**Training and mentorship**	
Poor quality of pre-service clinical training	Inadequate pre-service clinical supervision
	Lack of support during clinical practice
	High number of students in clinical practice
Health care providers’ continuing training system	Inadequate training opportunities
	Poor quality of in-service training
	Turnover of a trained provider
Mentorship and supervision	Lack of regular mentorship and supervision
	Lack of monitoring and evaluation

### Perceived benefits of following ANC guideline during clinical practice

#### I) Providers’ workload and performance

In this section, we describe the benefits of following the ANC guideline that improve the providers’ performance and have a positive effect on workload (Table 1).

*Impact on time*. Participants said that following guideline reduces the time they had to spend on a particular client by focusing on what should be done such as assessment, health promotion and care provision. Those with the most positive view of following the guideline said that it became easier to provide care. Indirectly it decreases the workload by focusing on the must-do activities as per the guideline. However, others who had a less positive view said that following the guidelines is very tedious and time-consuming.

*“The guideline guides me what must be done in every step. I will not waste time on what should be done next. So it saves my time to host more clients per day.”(Participant 5)*

*Structures the care activities*. Participants noted that following the ANC guideline carefully helps them to do activities sequentially from start to finish during the process of client evaluation and care provision.

“*If you used guideline*, *it will guide you on what to do at the beginning*, *in the middle*, *and at the end of client care*.*” (Participant 7)*

They also reported that they use the guideline document as a reference manual. They will do every activity by citing the guideline as a standard book.

Health care providers stated that following the guideline carefully was the best way to ensure that every step of history taking, physical examination and counseling is done during the ANC session.

*“……without a shadow of a doubt, having a guideline will help me to visualize my path and what should I do; otherwise I might miss important services.”(Participant 9)*

They also stated that poorly-timed and sub-standardized care might harm the woman and her fetus. Therefore, the more carefully the guideline is followed, the greater the minimization of medical errors.

*Increases competence among the providers*. Providers acknowledged that ANC is one of the core competencies of midwives. Practicing the ANC service regularly as per the guidelines makes the midwives more competent and proficient. Besides, they pointed out that carefully following the ANC guideline helps them to become more confident in front of clients and to be sure to deliver the service without feeling frustrated.

The participants also highly acknowledged that working with the ANC guideline during their day to day sessions supports the providers in making decisions about diagnosis and treatment of pregnancy-related complications without delay.

*“Measuring blood pressure alone does not help to diagnose hypertension during pregnancy. It has to be interpreted as per the guideline.”(Participant 2)*

#### II) Improvements in service delivery

In this section, a summary of the benefits of using the ANC guideline in improving service delivery is presented (Table 1).

*Ensuring the standard of the care*. Almost all participants acknowledged that they understood that the ANC guideline had been developed on the basis of high-level scientific research. Providing service based by following the guideline helps to ensure the provision of standardized, up-to-date, and evidence-based care.

In most of the interviews, participants noted that using ANC guideline helps them to provide complete and uniform care for all clients by all providers.

*“The guideline makes professionals speak the same language. In the absence of an experienced midwife, a junior can deliver the same ANC service alone if guideline is properly in place.”(Participant 6)*

*Enhance women-friendly care*. Study participants noted that following ANC guideline creates an opportunity to provide individualized and women-friendly care by putting women in the center of the care. They also reported that following the guideline helps to create a smooth client-provider relationship by involving the clients themselves in discussions and decisions.

*Improves continuum of care*. Most of the participants perceived that following the guidelines during ANC session increases service uptake by reducing extra time spent with each client. The participants also thought that improving the quality of the first meeting with quality of care to the pregnant women is likely to increase the motivation for the woman to attend subsequent recommended ANC visits.

*“Without any reservation, when our clients are satisfied and happy by the quality of our service provided in the first visit, they will be eager to comeback the second visit.”(Participant 1)*

Some healthcare providers noted that improving the quality of counseling on birth preparedness in every visit as per the guideline increases the awareness of pregnant women of the importance of institutional delivery and help those to agree give birth at the health facility.

*Improve the process of the care*. Participants perceived that strict adherence to ANC guideline during the first visit helps to improve the process of care through proper history taking, physical examination, and routine laboratory investigation by using an integrated ANC sheet. Then clients can be placed in the proper risk category based on their level of risk. Clients with low risk of pregnancy-related complications will need only universal routine care, and higher risk patients can be led to be given specialized care. Participants also acknowledged that following the guideline helps them to provide specific and comprehensive counseling services to promote and prevent complications during pregnancy.

*“When my client comes for the first time, I will ask her whether she had an abortion, previous stillbirth, and low birth weight. Once I identify she had bad obstetric history, I will provide specialized care because past bad obstetric outcomes are recurrent.”(Participant 5)*

*Maintaining the quality of service provision*. All of the participants had positive perceptions of the benefits of using the ANC guideline to improve the quality of the service they provide.

All participants expressed positive thoughts about the benefits of following ANC guideline as leading to greater client satisfaction. When pregnant women are satisfied with the quality of the service at the first visit, they will feel happy and eager to attend subsequent visits.

*“It is clear that when I provide ANC as per the protocol*, *my client feels happy because she perceived that she is getting the right care and she will develop trust in both the service and me as well*.*” (Participant 8*)

#### III) Improves perinatal outcomes

In all parts of the interview, participants reported that using the ANC guideline carefully would lead to more positive pregnancy outcomes for both the mother and the fetus and facilitating institutional delivery.

*“…………there is a usual principle in ANC practice*. *If you are providing the quality of care in every ANC contact*, *stillbirth to early neonatal death ratio should be comparable*. *If the stillbirth to early neonatal death ratio increases*, *our ANC service is sub-standard and compromised*.*” (Participant 9*)

*Perceived constraints that may limit complete adherence to ANC guideline*. Here we list major bottlenecks that may hinder complete adherence to the ANC guideline (Table 2).

#### 1) Organizational factors

In this section, institutional constraints that hinder the full implementation of ANC guideline by the providers are described.

*Unavailability of guideline*: *Overarching challenge*. Even though the participants identified many challenges to implementing ANC guideline, the single most important problem identified was that the original guideline was not even available to begin within their working places and this was therefore identified as an overarching challenge.

The participants said that they used an integrated ANC sheet prepared by the Ethiopian ministry of health. The sheet was extracted from the focused ANC guideline but was lacking details and depth on the contents of the services.

Participants also strongly noted that the ANC guideline document that had been given was not up to date. They said that the WHO 2016 eight-contact guideline had not been made available at all locations in Ethiopia. They said a guideline document with date of approval must be available throughout the country and must be posted on the bulletin board of all ANC rooms.

In some teaching health facilities, there is no culture of even using any ANC guideline document rather preferred to use international books. This may be related to differences in understanding of the importance of guidelines on the part of those who teach.

*“Some of the providers perceive that the American Confederation of obstetrician and gynecologists’ recommendations are similar to the Ethiopian ministry of health ANC guideline.” Participant 9)*

*Human resource-related factors*. The majority of the participants stated that the ratio of the number of clients to the number of providers is far too high. Many said they might encounter 40–50 clients at the health centers and 100–200 mothers in the hospital daily. If the interview of each client is supposed to take place in 20 minutes or less, then it may be impossible to follow even the ANC guidelines completely. If this is true, then the providers are faced with an impossible situation and apparently must choose which guideline items they will deal with. This may limit their ability to establish a good rapport with the women, provide adequate counseling, and avoid the need to make additional appointments on another day.

*“To evaluate a single client, it takes more than 15–20 min. So to give service for all clients, I might be in a rush by exempting essential ANC packages.” (Participant 7)*

The participants also stated that they suffer from a heavy workload and feel overburdened in trying to deal with the high client flow due shortage of human power. Health care providers working in the health centers are expected to deliver comprehensive MCH services such as ANC, delivery care, postpartum, and family planning units. This forces them to make compromises that mean they cannot adhere completely to the ANC guideline.

*“In our health center, there are three midwives who run MCH. One is working on a family planning service, so there are two remaining. One will be on duty and stay home the next day. So, one midwife will cover all services alone.”(Participant 3)*

Some providers said that there is no critical shortage of human power but that the problems arise due to poor management of the system. There are more than 30 temporally and permanently assigned health care providers including midwives, medical interns, and senior obstetricians. There is a mismanagement of human power. Senior consultants are not usually actively involved in the management of high-risk clients who need advanced care. Midwives are also not well involved in the decision-making process. So the majority of the workload is borne by medical interns who are not fully authorized to provide patient care.

*Lack of appropriate facility set up*. Participants observed that health facilities are not designed and constructed to make it possible to accommodate the number of clients who must be met. They stated that there are not enough rooms available for carrying out ANC interviews. With the exception of the hospital, there is only a single ANC room in each health center, and the rooms are so small that it is difficult to provide all services at the same time.

*“…… you can look at this, the only ANC room, which is too narrow to host many clients at the same time. With this narrow room, there are only two tables. So you cannot talk here about client privacy.” (Participant 3)*

They also stated that there is no separate MCH laboratory in most public health facilities. This increases the waiting time of clients due to delays in laboratory results. They recommend that a national standard be set for ANC rooms and efforts be made to meet this standard wherever possible.

All of the participants noted that power supply is a critical resource need for doing basic laboratory tests. There is an electric system in all health facilities but there is no continuous power supply due to a shortage of power sources at the national level. To overcome this problem, the majority of health facilities have backup generators.

*Logistic-related factors*. Participants noted that supplies and equipment need to be taken into consideration in setting ANC guideline. If the ANC guideline is to be followed, all materials specified by the guideline must be available especially for the first visit. The participants said there is at least one critical shortage of one or more of the following: health commodities, equipment, instruments, laboratory reagents and tests.

*“Since August 2019, we did not do hematocrit for our clients due to shortage of capillary tubes in our health center.”(Participant 3)*

The participants also noted that the major reasons for a critical shortage of essential supplies and equipment are: problems in the purchasing process, lack of an accountable body, and lack of communication between levels. In the procurement process, there is usually a failure to purchase functional and standard products such as blood pressure apparatus.

*“Now a day’s, these blood pressure apparatuses are not functional since it purchased. There was a clear problem with the quality of equipment during the procurement process.”(Participant 6)*

#### 2) Care providers’ knowledge, attitude, and skills

Below is a summary of care providers’ knowledge, attitudes, and skills identified as constraints, hindering complete adherence to ANC guideline.

*Poor providers’ attitude and behavior*. The participants stated that there is a lack of a culture of use of guidelines during clinical sessions. They said that they usually preferred to use the standard books as the basis for client care rather than national clinical protocols.

They also noted that they are not well-motivated and are reluctant to use ANC guideline in day to day clinical sessions for a variety of reasons: lack of recognition, feeling overburdened, and lack of incentives.

*“Our salaries are too small to cover our daily expenses and house rents. There is no extra incentive to drive our motivation. But we are trying our best to help our clients.”(Participant 6)*

*Knowledge and skills of providers*. Participants said that there is a variation in providers’ knowledge of the ANC guideline. Some of them stated that some of the items of the guideline, for example the one concerned with provision of iron and another about performing abdominal examination shouldn’t be followed in the first trimester but should be postponed to the second trimester when the pregnancy is apparent in the abdomen.

*“I don’t perform an abdominal examination when the client comes before 12 weeks of gestation. Why I am going to palpate the abdomen for the fetus already in the pelvis?”(Participant 5)*

The participants noted that two reasons for a variation in the implementation of ANC guideline are lack of awareness and lack of the necessary skills. Newly graduated midwives and medical interns don’t strictly adhere to local protocols due to a lack of prior clinical experience.

#### 3) Training and mentorship

The factors in the areas of training and mentorship that were identified as influencing complete adherence to ANC guideline are described below.

*Poor quality of pre-service clinical training*. The participants stated that less than ideal quality of pre-service clinical education significantly contributes to poor implementation of ANC guideline in some of the study health facilities. They stated that two health facilities, one specialized hospital, and one health center, were the parts of teaching health facilities for medical and midwifery students. Final year midwifery and medical interns are expected to conduct ANC sessions under the supervision of their clinical instructors. But due to the high number of students and loose clinical supervision, they struggled to implement the ANC guideline consistently.

*Health care providers continuing training system*. The participants noted that there are problems with the in-service training system in helping providers stay up-to-date and this also keeps away providers from adhering to ANC guidelines. They stated that ANC is one of the core competencies that all providers, particularly, midwives should have. Skill competency can be lost if there is no regular program of training to bring providers up-to-date. They perceived that there are inconsistencies in implementation due to differences in knowledge and skills as concerns the guideline. Therefore, refreshment training would help to maintain uniformity of practice during ANC practice.

Participants highlighted that training opportunities are scarce particularly in the area of ANC. There were BEmONC training programs given by non-governmental organizations but these did not reach most of the midwives who are directly involved in services to users. Those who attend the training sessions usually leave the ANC units due to different reasons such as changing in rotation and working place.

*Mentorship and supervision*. Participants identified the lack of supervision and mentorship as one of the major barriers to implement ANC guideline successfully. They indicated that there is no planned and structured supervision from the district or zonal health offices. Providers also perceived that they are unfairly blamed and insulted when service goals are not reached without technical support and feedback. They also perceive that planned supportive supervision and training would be useful to capacitate their skills, as well as for monitoring purposes.

*“I don’t think there is a body that evaluates the health center, no one has come asking us to fill our gaps.”(Participant 6)*

## Discussion

This study gained insights into participants’ perceptions of benefits and constraints of complete adherence to ANC guideline. We found that progressive incremental improvements in providers’ performance, provision of safe motherhood; and improvements in service delivery are the perceived benefits of following ANC guidelines. In agreement with this, previous research reported that the potential benefits of using guidelines in clinical settings include improving client outcomes, ensuring uniformity of care, improving the process and structure of care, and helping providers to make clinical decisions [4–8].

The results of the present study show that following the ANC guideline can save time, a definite benefit, but also that carrying out the interviews as prescribed takes too much time and this is a constraint. These statements are somewhat contradictory but none of the participants explained this contradiction. The majority of participants agreed that the Ethiopian ANC guideline is a well structured document that ideally should help them to be focused and concentrated on what should be done. Guidelines can save time if the provider does not have to spend time trying to think about what should be done. But the contrary view held by some was that following ANC guideline is very tedious and time-consuming. They said that the guidelines call for doing more than is reasonable in the time available given and the high client load. Therefore, full adherence to the guideline would result in a decrease in the number of clients who could be seen per day.

Although participants acknowledged the benefits of using ANC guideline, their level of adherence to national ANC guideline during first visit as it was surprisingly low at the four study facilities [27]. Through our findings, we have learned more from the participants themselves about their reasons for not following the guideline. Identifying barriers would help leaders and healthcare providers to create strategies for implementation of ANC guideline. A number of contributing factors were identified in this study including factors related to organization, health care providers, and training and mentorship.

Organizational problems were the leading and most important barriers that hinder complete adherence to the ANC guidelines. Among these barriers, the most frequently identified and overarching challenge during the clinical practice was the ANC guideline itself such as its format, contents, and availability. The original focused ANC guideline is not available physically during clinical practice. There is an integrated ANC sheet format used as a guideline. However, providers perceived that the format is lacking details and depth by content. A similar result has already been reported from other mixed study done in Jimma, Ethiopia [23]. Therefore, the ANC guideline should be available and modified in practical, simple, and easy-to-follow flowcharts that users could refer to quickly. Interestingly, the availability of clinical practice guideline on the internet by using mobile phones was found to be an important facilitator which is in line with other studies [33]. The other organizational problem that fuels poor adherence to ANC protocol was logistics-related problems. The implementation of the ANC guideline depends upon the availability of a large package of specific supplies and equipment [11]. Regarding the availability of basic equipment and supplies for focused ANC services, almost all study health facilities had scarcities but more worsen at the health centers. Interruption of power supply also made laboratory services less functional in all study facilities. This finding was supported by other studies done in Addis Abeba and Jimma [23, 34]. This would cause frustration of health care providers to provide ANC service as per the standards written in the guidelines made clients less satisfied with the service.

In our current study, ANC service at health centers was primarily delivered by midwives. The number of midwives with client ratio was not proportional. Midwives were quite busy in covering other MCH services simultaneously. This made them to exempt some important ANC services like counseling due to scarcity of time. This often causing midwives burnout and poor performance [35]. On the other hand, ANC at specialized hospital is given jointly by obstetrician, midwives, and Medical Interns. Obstetricians gave more attention for teaching of students than provision of clinical service. This finding is supported by other studies done Jimma by Villadsen et al [23] that showed a huge impact on implementing ANC guideline and contributed to the loss of continuity of care and client privacy.

Care providers’ knowledge, attitude, and skills were identified as constraints that hinder providers to follow ANC guideline. Findings from our study are consistent and supported by the other studies done in different settings [10, 11]. Poor providers’ attitude and behavior were the most frequently identified barrier to use and full implementation of ANC guideline. However, when we looked at the implementation of the guideline, one of the practical challenges is bringing about change in providers’ behavior. Changing attitudes and influencing perceptions is difficult [36]. Providers who lacked motivation and commitment toward the use of clinical practice guideline or those who were resistant to change were less likely to use clinical practice guidelines than providers who understood the reasons presented by WHO and others [37]. Surprisingly, in the current study, participants said that some of them are not well motivated and reluctant to use guideline in day to day clinical sessions due to different reasons. One of the identified reasons to smash their motivation is the lack of balanced financial compensation for their effort. Their salary wage is considered too small. This implies that the government should consider designing a strategy to motivate professionals.

Variations in participants’ knowledge of the contents of ANC guideline coupled with skill incompetency are the other barriers for complete adherence to ANC guideline. Some participants said that when clients come in the first trimester of pregnancy, they did not bother to provide some ANC services such as iron tablet supplementation or doing abdominal examination. This finding was supported by the study done in Mozambique, Ghana, and Bangladesh [11, 38, 39]. The major reasons for variations in implementing ANC guideline timely could be a lack of awareness on available guidelines and lack of clinical experience particularly medical interns. In contrast with the findings of our study, a study done on nurses with more work experience showed that they felt a high level of autonomy and were less likely to use clinical practice guideline than did junior and inexperienced nurses [40].

Also as evident from our findings that the quality of pre-service training is the other bottleneck that hinders provider’s adherence to ANC guideline. In teaching health facilities, medical interns are responsible to provide routine ANC service with little experience and competency. Senior obstetricians are not committed enough to coach their students while they are working in the clinical area. They are simply theoreticians at morning sessions. Midwives are not fully authorized to supervise medical students. This finding is consistent with another study done in Ethiopia [41] and one in Sierra Leone [42]. Therefore, integration of pre-service training to the service has to be done if there is to be better adherence to ANC guideline.

Research shows that short and competency-based ‘in-service’ training in emergency obstetric care results in significant improvements in healthcare provider competence and a move to making better use of guidelines [43]. In addition, training at the start of implementation and continuing education throughout the implementation process is to be recommended to increase the use of guidelines [44]. Midwives and Obstetricians identified lack of up-to-date training opportunities as a constraint that hinders the full implementation of guideline use in our present study which is concurrent with a previous study done in Ethiopia [23] and Mozambique [11]. Participants reported differences in knowledge of existing ANC guidelines due to the dynamicity of evidence at time advances This shows that the government has to design a continuous professional development system to improve health care professionals’ competency to prepare them to use the WHO eight-contact guideline. In addition to in-service training, supportive, planned, and periodic mentorship and supervision are recommended to increase adherence of health professionals to protocols by improving healthcare providers performance [45–47]. As consistent with other studies, care providers identified that there is too little supportive supervision and periodic mentorship [11]. The main challenges with supervision, also evident in the results of this paper, include improving the quality of supervision and increasing the time supervisors spend with healthcare providers.

To the best of our knowledge, this is the first qualitative research in Ethiopia to explore the reasons for not following ANC guidelines, therefore, the findings of our study can be *transferred* to other settings and situations that are similar. During analysis, all investigators were involved; discrepancies in the coding were discussed and a consensus was found. This increases the *credibility* of the research findings.

The study has some limitations. Although we have achieved the data saturation early, the generalizability (transferability) of the results from interviews with small sample size to the larger population could be compromised. All interviews were conducted by the investigators and this may have introduced some bias however transcription was done by professional colleagues who have tremendous experience in a qualitative study. Providing ANC service by inexperienced medical interns was one of the identified gaps during the interview. Therefore, it would have been nice to include them to be a part of the study because they might have their reasons for not following the ANC guideline. Finally, we were also not able to explore the constraints of implementing ANC as per the standards from the client’s perspective.

## Conclusions

Although participants acknowledged the benefits of using ANC guidelines during clinical practice, they themselves have not completely adhered to the existing Ethiopian ANC guideline. We identified provider, organizational and training and mentorship related groups of factors for the reasons why providers are not following ANC guideline during clinical practice. Attitude, knowledge and skills were identified as provider-related factors whereas clinical practice guideline, resources (human and material), and facility set up were the organizational-related factors. And also the quality of pre-service clinical training, continuous in-service training, and mentorship were training and mentorship-related factors. Based on these findings, we suggest a more comprehensive and focused strategy to address the challenges that midwives and obstetricians face challenges to provide ANC as per the standards. Primarily, the Ethiopian government should start to update and implement ANC guideline. Refresher training has to be given at the start of the adapted eight-contact ANC guideline and continuing education and supervision throughout the implementation process. Health care providers call for profound and urgent revisions to the supply chain system for supplies and equipment.

## Supporting information

S1 Text (DOCX)

## List of abbreviations

ANC: Antenatal Care
BEmONC: Basic Emergency Obstetric and Newborn Care
IRB: Institutional Review Board
LMIC: Low and Middle-Income Countries
MCH: Maternal and Child Health
WHO: World Health Organization
