# Supplementary material for: Provider-perceived benefits and constraints of complete adherence to antenatal care guideline among public health facilities, Ethiopia: A qualitative study
Source: PLoS One. 2021 Aug 9;16(8):e0255297. doi: 10.1371/journal.pone.0255297 (PMC8351951; doi:10.1371/journal.pone.0255297)
Supplement: S1 Text — (DOCX) [file pone.0255297.s001.docx]

| \| S1 Text: In-Depth Interview guide for health care providers \| \| --- \| \| Interviewee ID No. ____________  Gender: Male / Female  Name of health facility:___________  Date: _________________  **Introduction**  I am ______________________________ from ______________________   - General purpose of the study - Aims of the interview and expected duration - Who is involved in the process (other participants) - Why the participant’s cooperation is important - What will happen with the collected information and how the participant/target group will benefit - Any questions? - Consent   **Warm up [demographic & work history]**  Can I ask some details about you and your job?  How old are you? _________ years  Job Title ____________________________  What is your educational level?_________  From where did you get graduated?  Years worked at this facility ____________________  How long you work at ANC clinic? ____________  **Now I am going to ask you some questions about the benefits and constraints of following ANC guideline**   1. Have you ever heard about Guidelines? 2. What is a guideline? How can you explain it? Let us discuss about it. 3. What guidelines do you use in maternal and child health? 4. What is the benefits of following antenatal care guideline during clinical practice   **Probe:** benefits for the provider, maternal/fetal/neonatal/community   1. Do you think that antenatal is conducted as per the guideline in your institution? 2. If no, What are the constraints to use ANC guideline in your facility   **Probe:** challenges related with organization (guidline,equipement and supplies, facility set up , human resource, client load, working environment), provider related( knowledge skill and attitude, and skill) , capacity building (in-service and pre-service training), mentorship and policy at large   1. How do you explain the intra and inter personal relationships among health care professionals in applying the antenatal care guidelines   **Probe**- do you think intra and inter personal relationships among health care professionals affect utilization of ANC guidelines? If yes or no, why and how?   1. What things should be improved in the future to implement ANC guideline in your institution effectively?   **Closing**   1. Is there anything else you want to add on the benefits and constraints following ANC guideline that we have not talked about? 2. Summarize 3. Thank s for your participation \|   I have discussed the study with the respondent signed above, in a language he/she can comprehend.  I believe he/she has understood my explanation and agrees to take part in the interview.   \| **NAME**  (in capital letters) \| **SIGNATURE** \| **DATE OF SIGNATURE**  (in DD/MM/YYYY) \| \| --- \| --- \| --- \| \|  \|  \|  \| |
| --- | --- | --- | --- | --- | --- | --- | --- | --- |

| **ለጤና ባለሙያዎች ጥልቅ ቃለ መጠይቅ መመሪያ** |
| --- |
| **የመረጃ ሰብሳቢዉ ፊርማ :** |
| ከላይ ካአስፈረመኳት የጥናቱ ተሳታፊ ጋር በሚያግባባን ቋንቋ በደንብ ስለጥናቱ አላማ ተነጋግረናል፡፡, የነገርኳትን ተረድታኝ የጠናቱ አካል ለመሆን የተስማማቸ መሁኑንተረድቻለሁ፡፡.   \| **ስም**  (in capital letters) \| **ፊርማ** \| **የፈረመበት ቀን**  (DD/MM/YYYY) \| \| --- \| --- \| --- \| \|  \|  \|  \| |

| የተሳታፊዉ መለያ ቁጥር ____________  ፆታ: ወንድ / ሴት  የጢና ተቋሙ ስም:___________  ቀን: _____________  **መግበያ**  እኔ ___________________ ነኝ______________________ ከጎንደር ዩኒቨርሲቴ   - የጥናቱ ጠቅላላ አላማ - የቃለ-ምልልሱ ዋና አላማና የሚዎስድበት ጊዜ - ጥናቱ ዉስጥ እነ ማን ይሳተፋሎ - የተሳታፈዎች ትብብር ለምን አስፈለገ - የጥናቱ መረጃዎች ለምን ይሰበሰባሉ፤ ከዚህ ጥናት ተሳታፊዎች ከጥናቱ በምን መንገድ ይጠቀማሉ፤፤ - ጥያቄ? - ፈቃደኝነት   **ማማቂያ**  **ሰለ አነተ/ች አና ስለ ስራሸ አንዳነድ ጥያቂዎች መጠየቅ እችላለሁ**   - እደሜህ/ሸ /ህ ሰንት ነዉ - የስራ መአረግሸ/ህ ____________________________ - የትመህርት ደረጃሸ/ህ ?_________ - ከዚህ ጢና ተቋም ስነት አመት ሰራሸ/ህ ______________(በወራት) - ከዚህ ነብሰ-ጡር ክትትል ክፍል ከሰራሸ/ህ ሰንት አመት ሆነሸ/? ________(በወራት)   **አሁን በዚህ ጢና ተቋም ያለህን የስራ ሁኒታ በተመለከተ አንዳነድ ጥያቂዎችን አንነጋገራለን**   1. ስለ መመሪያዎች ሰምተህ ታውቃለህ? 2. መመሪያ ምንድን ነው? እንዴት ልታብራራው ትችላለህ? ስለዚህ ጉዳይ እንወያይ ። 3. በእናቶችና በህፃናት ጤና ላይ የትኞቹን መመሪያዎች ትጠቀማላችሁ? 4. በክሊኒካል ልምምድ ወቅት የቅድመ ወሊድ ህክምና መመሪያ መከተል የሚያስገኛቸው ጥቅሞች ምንድን ናቸው   Probe: ለጤና ባለሙያ /የእናቶች/የወሊድ/ አዲስ ለተወለደ ልጅ /ማህበረሰብ እይታ ጥቅሙ   1. ቅድመ ወሊድ በእርስዎ ተቋም ውስጥ ባለው መመሪያ መሰረት የሚከናወን ይመስላችኋል? 2. አይደለም ከሆነ በተቋምዎ ውስጥ የቅድመ ወሊድ እንክብካቤ መመሪያ ለመጠቀም የሚገደቡት ገደቦች ምንድን ናቸው   Prob: ከአደረጃጀት (መመርያ፣ መሣሪያና እቃዎች፣ ተቋማት የተቋቋሙ፣ የሰው ሀብት፣ የደንበኞች ጫና፣ የሥራ አካባቢ)፣ የጤና ባለሙያ ተዛማጅ (የዕውቀት ክህሎትና አመለካከት፣ እና ክህሎት) ፣ የአቅም ግንባታ (በአገልግሎትና ቅድመ-አገልግሎት ሥልጠና)፣ ክትትል ማስተማሪያና ፖሊሲ በአጠቃላይ   1. የቅድመ ወሊድ እንክብካቤ መመሪያዎችን ተግባራዊ በማድረግ ረገድ በተለያዩ የጤና ባለሙያዎች መካከል ያለውን የውስጥ እና እርስ በርስ የግል ግንኙነት እንዴት ታብራራለህ? 2. በተቋምዎ ውስጥ የቅድመ ወሊድ መመሪያን ውጤታማ በሆነ መንገድ ለመተግበር ወደፊት ምን ነገሮች መሻሻል አለባቸው?   **መዝጊያ**   1. ያልተነጋገርነውን የቅድመ ወሊድ መመሪያ መከተል በሚያስገኛቸው ጥቅሞችና ገደቦች ላይ መጨመር የምትፈልገው ሌላ ነገር አለ? 2. ማጠቃለያ 3. ለተሳትፎሸ/ህ በጣም አመሰግናለሁ |
| --- |
